# Development and Validation of a Nomograph Model for Post-Operative Central Nervous System Infection after Craniocerebral Surgery

**DOI:** 10.3390/diagnostics13132207

**Published:** 2023-06-29

**Authors:** Li Cheng, Wenhui Bai, Ping Song, Long Zhou, Zhiyang Li, Lun Gao, Chenliang Zhou, Qiang Cai

**Affiliations:** 1 Department of Critical Care Medicine, Eastern Campus, Renmin Hospital of Wuhan University, Wuhan 430200, China; rm003651@whu.edu.cn; 2 Department of Hepatobiliary Surgery, Eastern Campus, Renmin Hospital of Wuhan University, Wuhan 430200, China; 3 Department of Neurosurgery, Eastern Campus, Renmin Hospital of Wuhan University, Wuhan 430200, China

**Keywords:** neurosurgery, post-operative central nervous system infection, nomograph

## Abstract

Purpose: A nomograph model of predicting the risk of post-operative central nervous system infection (PCNSI) after craniocerebral surgery was established and validated. Methods: The clinical medical records of patients after cranial surgery in Renmin Hospital of Wuhan University from January 2020 to September 2022 were collected, of whom 998 patients admitted to Shouyi Hospital District were used as the training set and 866 patients admitted to Guanggu Hospital District were used as the validation set. Lasso regression was applied to screen the independent variables in the training set, and the model was externally validated in the validation set. Results: A total of 1864 patients after craniocerebral surgery were included in this study, of whom 219 (11.75%) had PCNSI. Multivariate logistic regression analysis showed that age > 70 years, a previous history of diabetes, emergency operation, an operation time ≥ 4 h, insertion of a lumbar cistern drainage tube ≥ 72 h, insertion of an intracranial drainage tube ≥ 72 h, intraoperative blood loss ≥ 400 mL, complicated with shock, postoperative albumin ≤ 30 g/L, and an ICU length of stay ≥ 3 days were independent risk factors for PCNSI. The area under the curve (AUC) of the training set was 0.816 (95% confidence interval (95%CI), 0.773–0.859, and the AUC of the validation set was 0.760 (95%CI, 0.715–0.805). The calibration curves of the training set and the validation set showed *p*-values of 0.439 and 0.561, respectively, with the Hosmer–Lemeshow test. The analysis of the clinical decision curve showed that the nomograph model had high clinical application value. Conclusion: The nomograph model constructed in this study to predict the risk of PCNSI after craniocerebral surgery has a good predictive ability.

## 1. Introduction

PCNSI refers to the infection caused by the invasion of various pathogenic microorganisms into the central nervous system due to trauma, edema, hemorrhage, stress, and other causes after craniocerebral surgery. PCNSI is one of the serious complications of neurosurgery, which may lead to a second operation, increase mortality and hospitalization costs, and even result in patients remaining with permanent sequelae [1,2]. Some studies have shown that the incidence of PCNSI is 4.6–25% [3,4], which accounts for 0.8–7% of central nervous system infections (CNSIs) [5]. However, the incidence of PCNSI varies with different hospitals, different diseases, different surgical methods, and different diagnostic criteria. Although the incidence of PCNSI has been reduced with the improvement of operating room equipment, the rapid development of neurosurgical techniques and the widespread use of prophylactic antibiotics [6], the drug resistance rate of pathogenic bacteria has increased year by year and the positive rate of bacterial culture has decreased due to the application of broad-spectrum antibiotics, which further increases the difficulty of treatment [7]. Therefore, accurate prediction of the risk of PCNSI is of great significance to guide the prophylactic use of anti-infective therapy in the perioperative period. The purpose of this study is to determine the risk factors of PCNSI and construct a nomograph model to guide the clinical decision-making of differentiated use of anti-infective therapy.

## 2. Materials and Methods

### 2.1. General Data

This study is a single-center (dual hospital district), retrospective, observational cohort study. The clinical data of 1864 patients after craniocerebral surgery in Renmin Hospital of Wuhan University from January 2020 to September 2022 were collected. The patients in Shouyi Hospital District were taken as the training set (*n* = 998), while the patients in Guanggu Hospital District were taken as the verification set (*n* = 866). Patients who were scheduled to undergo craniocerebral surgery upob admission were taken as the starting point of the study, and the occurrence of PCNSI within 30 days after craniocerebral surgery was taken as the end point of the study.

#### 2.1.1. Inclusion Criteria

① Patients admitted to the neurosurgery department or intensive care unit of our hospital for ≥7 days after initial craniocerebral surgery; ② age ≥ 18 years old; and ③ patients with complete case data.

#### 2.1.2. Exclusion Criteria

① Patients with initial craniocerebral surgery in another hospital; ② patients with CNSIs before craniocerebral surgery; ③ patients who died or were discharged from hospital within 7 days after the operation; ④ age < 18 years old; and ⑤ patients with incomplete data.

#### 2.1.3. Diagnostic Criteria for PCNSI

Those who met the clinical diagnosis or etiological diagnosis were included in the study with reference to the expert consensus [8].

##### Clinical Diagnostic Criteria

Patients with fever, intracranial hypertension, turbid or purulent cerebrospinal fluid (CSF), leukocytosis, glucose < 2.2 mmol/L, and CSF glucose content/serum glucose content ≤ 0.4.

##### Etiological Diagnostic Criteria

Patients with positive microbiological cultures of specimen smears, drainage tube tips, implants, and CSF on the basis of clinical diagnosis, excluding those with bacterial contamination and colonization.

### 2.2. Data Collection

**Figure 1:**
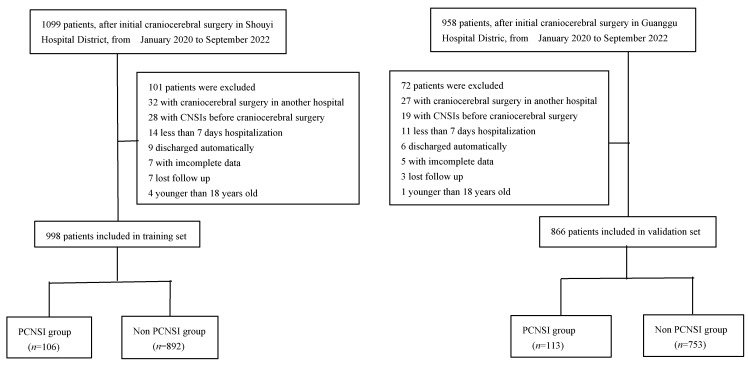
Flowchart of enrolled craniocerebral surgery patients in the training set and validation set.

Structured data extraction forms were administered by trained researchers for collection. The detailed flow chart of data collection is shown in Figure 1.

### 2.3. Statistical Methods

Statistical analysis and mapping were performed using SPSS 26.0 and R 4.1.3. None of the measurement data in this study followed the normal distribution, and measurement data that were not normally distributed were expressed as the median (quartile) [M (Q_L_, Q_U_)], and comparisons between the groups were performed using the Mann–Whitney U test. For count data presented as [*n*(%)], comparisons between the groups were performed using the Chi-square test or Chi-square test with continuous correction or the Fisher’s exact probability test. Whether PCNSI occurred within 30 days after craniocerebral surgery (secondary outcome index) was used as the dependent variable. The independent variables were screened by Lasso regression, and the risk factors were screened by multivariate logistic regression analysis. The R software RMS package was applied to construct the nomogram model, and the predictive ability of the model was evaluated by the ROC. The bootstrap method was used to repeat the sampling 1000 times for internal validation and to compare the difference in the C-index. Furthermore, the prediction model was evaluated using calibration curves and DCA. The difference was statistically significant when *p* < 0.05.

## 3. Results

### 3.1. Analysis of Baseline Data of Patients

In total, 106 (10.62%) of the 998 patients in the training set had a PCNSI, while 113 (13.05%) of the 866 patients in the validation set had a PCNSI. However, there was no significant difference in the incidence of PCNSI between the two groups (*χ*^2^ = 2.635, *p* = 0.105). The clinical baseline characteristics are shown in Table 1.

### 3.2. Lasso and Logistic Regression Analysis

**Figure 2:**
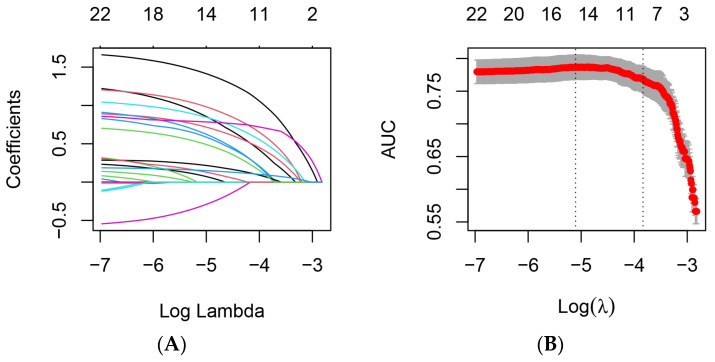
Relation curve between the penalty coefficient of the lasso regression variable and the log lambda (**A**); relation curve between the lasso regression AUC and Log(λ) (**B**).

A total of 44 potential risk factors associated with PCNSI were included in the study. The dimension of the training set variables was reduced by Lasso regression, and the most representative feature variables were selected. Five-fold cross validation was adopted in selecting the optimal lambda parameter, and the number of variables at this time was counted by taking the smallest lambda value in cross-validation error as the model optimal (see Figure 2). Each curve in Figure 2A represents the variation trajectory of a independent variable coefficient. The results of the lasso regression analysis showed that 14 independent variables were characteristic variables affecting PCNSI (Figure 2B). The results of the multifactorial logistic regression analysis with the occurrence of PCNSI or not as the dependent variable and the 14 characteristic variables selected by lasso regression as independent variables showed (Table 2) that 10 independent variables were the independent risk factors for PCNSI.

### 3.3. Establishment of Nomogram Model

**Figure 3:**
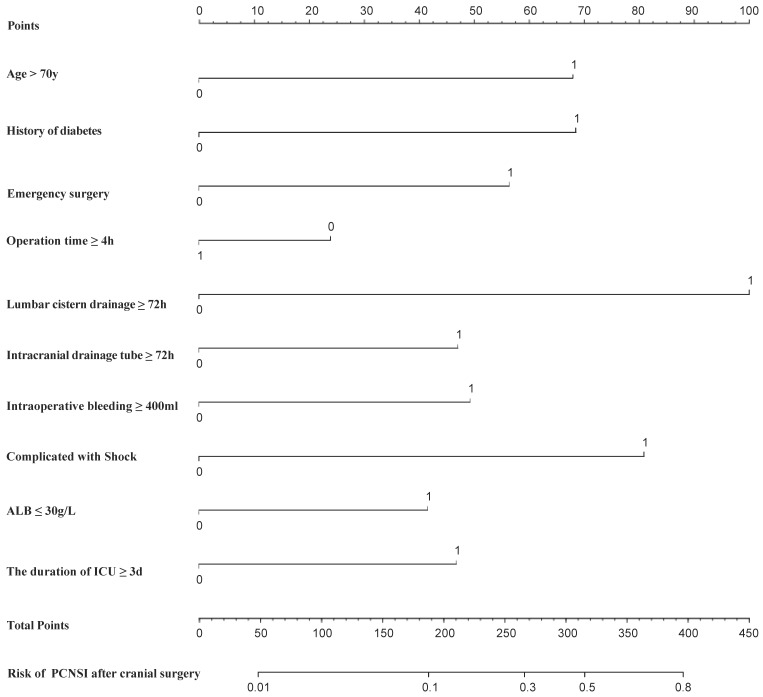
Construction of a nomogram model for PCNSI after cranial surgery.

The nomogram model was constructed based on 10 independent variables determined by multivariate logistic regression analysis (Figure 3). Clinicians could assess the risk of PCNSI in a visualized, individualized, and quantitative way based on these 10 easily available metrics. The C-index of the training set was 0.816 (95%CI, 0.773~0.859). The greater the C-index was, the better the differentiation of the model was, indicating that the accuracy of nomogram prediction was good.

### 3.4. Internal Validation of the Model

The C index of the training set was the same after the internal verification of the bootstrap, that is, the model prediction results were consistent with the real results.

### 3.5. External Validation of the Model

**Figure 4:**
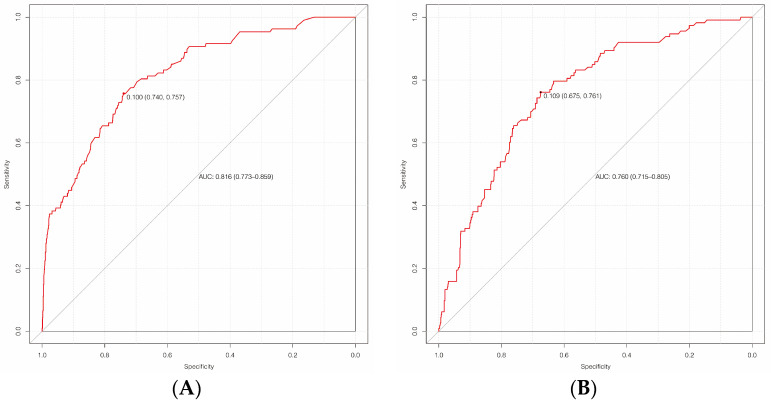
ROC of the nomogram predicting the risk of PCNSI after cranial surgery ((**A**) is the training set; (**B**) is the validation set).

**Figure 5:**
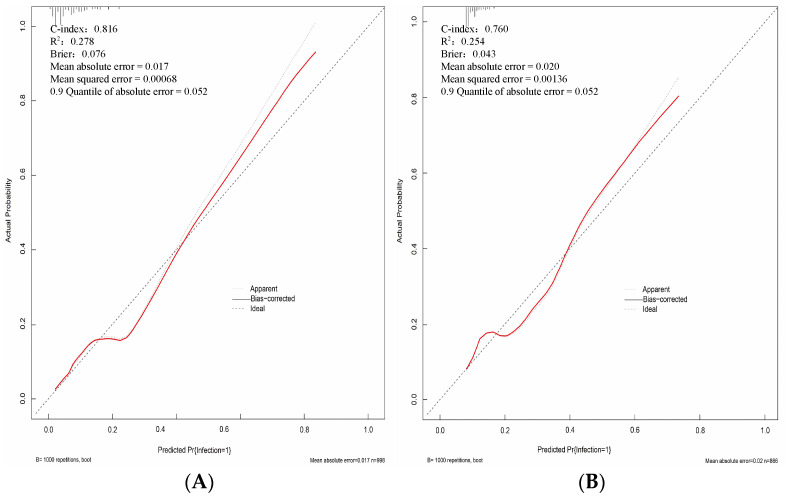
Calibration curve of the nomogram predicting the risk of PCNSI after cranial surgery ((**A**) is the training set; (**B**) is the validation set).

The AUC of the training set was 0.816 (95%CI, 0.773~0.859), with a sensitivity of 74.0% and a specificity of 75.7%, while the AUC of the validation set was 0.760 (95%CI, 0.715~0.805), with a sensitivity of 67.5% and a specificity of 76.1%, indicating that the prediction performance was good, as shown in Figure 4. The calibration curve was plotted to assess the discriminatory efficacy of the nomograph model, and a Hosmer–Lemeshow test was conducted. The calibration plots of the training and validation sets showed that the nomograph had a good fit with the reference line, as shown in Figure 5, with *p*-values of 0.439 and 0.561, respectively (all *p* > 0.05). The Brier scores were 0.076 and 0.043, respectively (all close to 0), indicating that the prediction of the probability of PCNSI infection after craniocerebral surgery by the nomograph model was consistent with the actual infection percentage of the observed population.

### 3.6. Clinical Decision Curve Analysis (Figure 6)

The DCA determined the value of the clinical application of the nomograph model by calculating the net benefit under the PCNSI risk threshold probability of each patient after craniocerebral surgery. The horizontal coordinate of the DCA was the threshold probability of high risk, and the vertical coordinate was the net benefit (NB). The probability of patients developing PCNSI was noted as P_i_ when the nomograph model reached a certain value, and when P_i_ reached a certain threshold, it was defined as positive (noted as P_t_). The high-risk threshold was set as (0, 1), and the net benefit rate and the range of effective pretest probability were assessed by subtracting the false-positive population that was incorrectly judged by the model. When all patients had no PCNSI or all of them had a PCNSI, the nomogram model had no clinical application value. The threshold probability of the training set was between 0.01~0.74 and that of the validation set was between 0.01~0.83, all of which had a net benefit rate >0 and had clinical practical value, which suggested that the model had good clinical value in predicting PCNSI after craniocerebral surgery.

**Figure 6:**
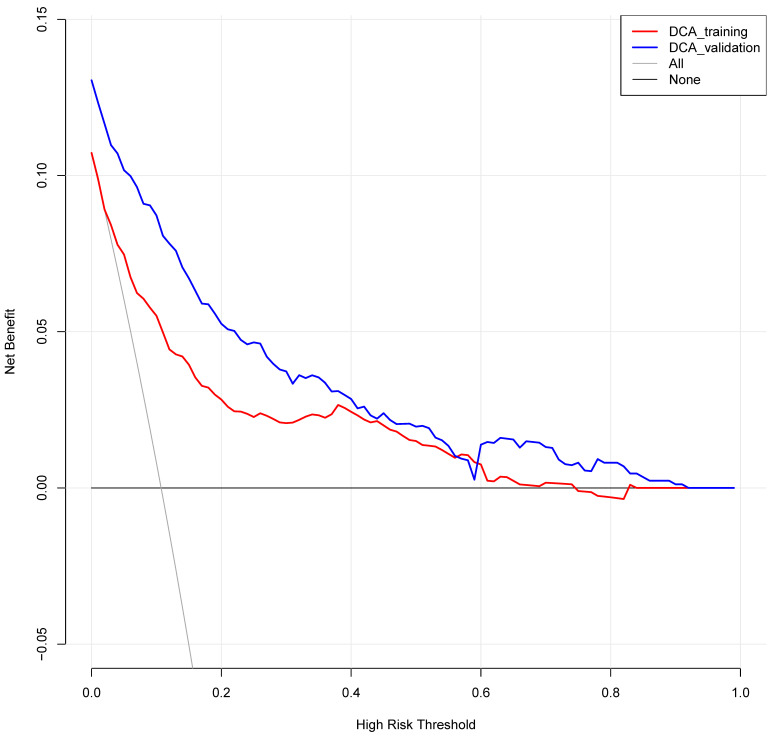
DCA of the nomogram predicting the risk of PCNSI after cranial surgery.

## 4. Discussion

PCNSI is the most serious hospital-acquired infection [9]. The case fatality rate of meningitis and (or) ventriculitis after craniocerebral surgery is as high as 3~33% [10], and even if patients with a PCNSI are cured, they will generally remain with varying degrees of neurological dysfunction, which seriously affects the prognosis and quality of life of patients. At the same time, the treatment of PCNSI is also one of the important clinical problems faced by neurosurgeons at present. There are limited antibiotics that can pass through the blood–brain barrier or reach a higher concentration in cerebrospinal fluid. In the absence of etiological evidence, the long-term use of broad-spectrum antibiotics will lead to the majority of bacteria becoming drug-resistant. However, the construction of a predictive model can screen out patients with PCNSI after high-risk craniocerebral surgery, and individualized prophylactic use of antibiotics during the perioperative period can improve the prognosis of patients [11].

Our study focused primarily on patients after craniocerebral surgery and developed and verified a practical model to identify people with high PCNSI risk. The development and validation of the model followed the requirements and recommendations in the tripod statement [12]. In this study, potential predictors were screened in detail, while those with more missing values and those that could not be generally detected in clinic were excluded. The model consisted of 10 variables, which could be easily obtained upon admission and during and after the operation. The model was externally validated in the validation set, with high discrimination and good prediction performance.

Previous studies have pointed out that advancing age is a risk factor for the development of PCNSI [13,14], which is consistent with the conclusion of this study. Patients with diabetes have a high risk of PCNSI, which may be due to the fact that hyperglycemia can inhibit growth factor synthesis, angiogenesis, collagen deposition, and fibroblast proliferation and migration, making the wound difficult to heal and providing favorable conditions for bacterial proliferation. This is consistent with the research conclusions reported in the literature [15,16]. This study found that the risk of PCNSI in patients undergoing emergency operations is high, which may be related to more open craniocerebral injury, and most emergency patients are in a critical condition and have a severe stress response, which is consistent with the conclusion of the study report [17]. However, it is different from the research results of Ren Xiaohui [18], which may be related to the different selection of the timing of surgical intervention. Operation time ≥ 4 h and intraoperative blood loss ≥ 400 mL are risk factors of PCNSI, which may be related to the air pollution caused by the exposure of surgical wounds and medical devices. At the same time, large wounds and more bleeding reduce systemic and local resistance to external pathogens, and patients are under anesthesia during operations and the body reduces the stress ability to external bacterial invasion [19], which is consistent with the findings of most studies [14,17,20,21]. Insertion of lumbar cistern and a intracranial drainage tube ≥72 h are high-risk operations of PCNSI, which may be related to retrograde infection of pathogens caused by delayed removal of the drainage tube, failure to strictly perform aseptic operation, and improper nursing, which is consistent with the results reported by X Huang [21] and contradicts the results reported by Y F Zhang [22]. It may be related to the main focus of this study on cerebrospinal fluid indexes. In a prospective multicenter study of 2944 patients with the risk of intracranial infection after neurosurgical craniotomy, Korinek et al. [23] found that CSF leakage was an independent risk factor for PCNSI, but no statistical difference was found in our study, which may be related to the low overall incidence (4.35%) of CSF leakage in this study. On this basis, our study paid more attention to the organ function and immunomodulatory factors, which led to the conclusion that shock and postoperative albumin ≤30 g/L were risk factors for PCNSI. Therefore, the importance of early screening is emphasized in order to provide individualized and precision treatment for patients. The length of stay in ICU ≥ 3 days is one of the risk factors of PCNSI, which may be due to the critical condition of some patients and their weak ability to resist the invasion of pathogenic bacteria, as well as the more complex pathogenic bacteria and more drug-resistant bacteria in the ICU [24]. In the previously published prediction models [11,22,25], PCNSI mostly considered the risk factors related to surgery or the operating room, while this study comprehensively considered the basic state of patients, disease severity, and complications.

The nomograph model constructed in this study can directly predict the magnitude of the risk probability of PCNSI. For example, a patient aged 75 years with a previous history of diabetes mellitus was admitted to the hospital for emergency surgery, with an operation time duration of 5 h, intraoperative blood lost amounting to 500 mL, a intracranial drainage tube placed for more than 5 d, no lumbar cistern drainage tube placed, no preoperative, intraoperative, or postoperative shock combined, a postoperative ALB of 28.8 g/L, and a 5-d ICU stay. The scores of each predictor variable were calculated according to the nomograph model as 68, 68, 58, 22, 44, 0, 50, 0, 42, and 48, respectively, with a total score of 400; then, the probability of PCNSI was more than 80%, which should be paid enough attention by clinicians. After evaluation of the prediction model, the following diagnostic and treatment decisions were made for this patient: 1. strengthen the management of hand hygiene in health care; 2. improve blood culture, cerebrospinal fluid culture, or the NGS of cerebrospinal fluid as soon as possible to clarify the pathogenic bacteria; 3. remove the intracranial drainage tube as soon as possible according to the condition; 4. timely supplementation of human serum albumin to ALB ≥ 30 g/L; monitor and control blood glucose; 5. transfer the patient out of the ICU as soon as possible when the patient’s condition was stable; and 6. according to the epidemiological characteristics of the pathogenic bacteria in the region and unit, vancomycin combined with meropenem, ceftriaxone, cefepime, and other bacterial meningitis systemic intravenous dosing regimens was empirically selected [26], and adjust the target therapy in the time after pathogenic return, and if the treatment effect was poor, it could be combined with ventricular or intrathecal injection [8].

However, our study still has limitations as follows. Firstly, it is a single-center (dual hospital district), retrospective, observational cohort study based on central China, which may not be representative of patients after cranial surgery in other countries or regions due to differences in racial, geographical, economic, and medical levels. Secondly, this study is a retrospective study, and the data derived from the electronic medical record system are not all complete; therefore, we tried to use the random forest method to reduce the bias caused by some missing data. Thirdly, we did not collect the follow-up antimicrobial treatment of the patients, and the influence of different treatment regimens was not considered in the nomograph model. Fourth, some indicators reported in the literature, such as CSF indicators, cranial imaging indicators, and cytokine indicators, have not been evaluated in the nomograph model due to missing data. Finally, the laboratory data may change with the progress of the disease, and it is impossible to include the dynamic changes of various indicators in the model for analysis due to the nature of the retrospective study.

## 5. Conclusions

The nomogram model established in this study has good predictive ability and discrimination. The model has high stability, reliability, and repeatability, which is worthy of clinical promotion and application.

## Figures and Tables

**Table 1:** Comparison of baseline data between the training set and the validation set.

Characteristic	Training Set (*n* = 998)	Validation Set (*n* = 866)	*Z*/*χ^2^* Value	*p*-Value
Male/female (cases)	530/468	464/402	0.042	0.838
Age [*n*(%)]				
18~40 years	260 (26.1)	216 (24.9)	0.300	0.584
40~50 years	128 (12.8)	129 (14.9)	1.672	0.196
50~60 years	252 (25.3)	241 (27.8)	1.276	0.259
60~70 years	150 (15.0)	129 (14.9)	0.007	0.936
>70 years	208 (20.8)	151 (17.4)	3.457	0.063
Comorbidities [*n*(%)]				
Hypertension	206 (20.6)	199 (23.0)	1.490	0.222
Diabetes	96 (9.6)	90 (10.4)	0.309	0.578
Infection in other parts	38 (3.8)	43 (5.0)	1.598	0.206
Autoimmune disease	26 (2.6)	12 (1.4)	3.453	0.063
Pathogenies [*n*(%)]				
Open craniocerebral injury	177 (17.7)	128 (14.8)	2.958	0.085
Closed craniocerebral injury	65 (6.5)	77 (8.9)	3.727	0.054
Hemorrhagic stroke	356 (35.7)	287 (33.1)	1.314	0.252
Ischemic stroke	61 (6.1)	45 (5.2)	6.338	0.012
Intracranial tumor	290 (29.1)	285 (32.9)	3.225	0.073
Others	49 (4.9)	44 (5.1)	0.014	0.905
Type of infections [*n*(%)]				
Epidural abscess	11 (9.7)	0 (0.0)	-	<0.001 ^a^
Subdural empyema	7 (6.2)	0 (0.0)	-	<0.001 ^a^
Meningitis	41 (36.3)	0 (0.0)	-	<0.001 ^a^
Ventriculitis	22 (19.5)	0 (0.0)	-	<0.001 ^a^
Brain abscess	32 (28.3)	0 (0.0)	-	<0.001 ^a^
Pathogen types [*n*(%)]				
G^+^	31 (27.4)	0 (0.0)	-	<0.001 ^a^
G^−^	35 (31.0)	0 (0.0)	-	<0.001 ^a^
Fungus	9 (8.0)	0 (0.0)	-	<0.001 ^a^
Type of surgeries [*n*(%)]			3.609	0.057
Emergency surgery	361 (36.2)	277 (32.0)	-	-
Elective surgery	637 (63.8)	589 (68.0)	-	-
Operation mode [*n*(%)]				
Craniotomy	35 (31.0)	259 (34.4)	0.513	0.474
Cranial burr-hole	47 (41.6)	296 (39.3)	0.214	0.644
Neuroendoscope	31 (27.4)	198 (26.3)	0.066	0.798
Operation time [*n*(%)]			1.877	0.171
≥4 h	222 (22.2)	216 (24.9)	-	-
<4 h	776 (77.8)	650 (75.1)	-	-
Intraoperative bleeding [*n*(%)]			1.297	0.255
≥400 mL	161 (16.1)	155 (18.1)	-	-
<400 mL	837 (83.9)	700 (81.9)	-	-
CSF leak [*n*(%)]	39 (3.9)	42 (4.8)	0.990	0.320
Intracranial drainage tube [*n*(%)]				
≥72 h	334 (33.5)	312 (36.0)	1.343	0.247
<72 h	328 (32.9)	304 (35.1)	1.036	0.309
Lumbar cistern drainage [*n*(%)]				
≥72 h	272 (27.3)	258 (29.8)	1.467	0.226
<72 h	216 (21.6)	158 (18.2)	3.339	0.068
After CPCR [*n*(%)]	50 (5.0)	52 (6.0)	0.887	0.346
Complicated with Shock [*n*(%)]	136 (13.6)	132 (15.2)	0.983	0.322
Mechanical ventilation time [*n*(%)]				
≥48 h	149 (15.1)	152 (17.6)	2.072	0.150
<48 h	200 (20.0)	156 (18.0)	1.232	0.267
Total parenteral nutrition ≥ 5 d [*n*(%)]	139 (13.9)	146 (16.9)	3.076	0.079
ALB ≤ 30 g/L [*n*(%)]	420 (42.1)	354 (40.9)	0.278	0.598
The duration of ICU [days,M(Q_L_, Q_U_)]	4.0 (3.0,5.0)	5.0 (4.0,6.0)	−8.393	<0.001
APACHE II score [points,M(Q_L_, Q_U_)]	13.0 (10.0,16.0)	13.0 (10.0,17.0)	−3.599	<0.001
GCS score [points,M(Q_L_, Q_U_)]	14.0 (8.0,15.0)	12.0 (6.0,15.0)	−4.116	<0.001

Notes: G^+^ is Gram positive; G^−^ is Gram negative; CSF is cerebrospinal fluid; CPCR is cardiopulmonary cerebral resuscitation; APACHE II score is the acute physiology and chronic health evaluation II score; GCS score is the Glasgow coma scale score; and ^a^ is the Fisher’s exact test.

**Table 2:** Multifactor logistics regression analysis of PCNSI after cranial surgery.

Variable	*Β*	*SE*	*Wald*	*p*	*OR*	95%*CI*
Age > 70 y	1.171	0.261	20.179	**<0.001**	3.225	1.935–5.375
History of diabetes	1.253	0.310	16.355	**<0.001**	3.502	1.908–6.429
APACHE II score	−0.002	0.027	0.007	0.931	0.998	0.946–1.053
GCS score	0.002	0.028	0.008	0.930	1.002	0.948–1.060
Emergency surgery	1.033	0.240	18.462	**<0.001**	2.808	1.753–4.498
Operation time ≥ 4 h	−0.610	0.295	4.284	**0.038**	0.543	0.305–0.968
Lumbar cistern drainage ≥ 72 h	1.739	0.304	32.710	**<0.001**	5.689	3.135–10.323
Intracranial drainage tube ≥ 72 h	0.949	0.241	15.513	**<0.001**	2.583	1.611–4.143
CSF leak	0.503	0.516	0.591	0.329	1.654	0.602–4.545
Intraoperative bleeding ≥ 400 mL	0.923	0.282	10.725	**0.001**	2.516	1.448–4.370
Complicated with Shock	1.080	0.296	13.328	**<0.001**	2.945	1.649–5.258
Total parenteral nutrition ≥ 5 d	0.324	0.330	0.964	0.326	1.383	0.724–2.641
ALB ≤ 30 g/L	0.769	0.238	10.421	**0.001**	2.158	1.353–3.442
The duration of ICU ≥ 3 d	0.199	0.047	17.839	**<0.001**	1.220	1.112–1.337
Constant	−5.637	0.698	65.226	0.000	0.004	--

Notes: APACHE II score is the acute physiology and chronic health evaluation II score; GCS score is the Glasgow coma scale score; CSF is cerebrospinal fluid; and ICU is intensive care unit. Bold represents emphasized and statistically significant *p*-values, while italics represent statistical parameters.

## Data Availability

The original contributions presented in the study are included in the article, and further inquiries can be directed to the corresponding author.
